# Analysis of the Effect of Machining of the Surfaces of WAAM 18Ni 250 Maraging Steel Specimens on Their Durability

**DOI:** 10.3390/ma15248890

**Published:** 2022-12-13

**Authors:** Daren Peng, Andrew S. M. Ang, Alex Michelson, Victor Champagne, Aaron Birt, Rhys Jones

**Affiliations:** 1 ARC Training Centre on Surface Engineering for Advanced Materials (SEAM), School of Engineering, Swinburne University of Technology, Hawthorn, VIC 3122, Australia; 2 Centre of Expertise for Structural Mechanics, Department of Mechanical and Aerospace Engineering, Monash University, Clayton, VIC 3800, Australia; 3 Solvus Global, 104 Prescott Street, Worcester, MA 01605, USA; 4 US Army Research Laboratory, U.S. Army Combat Capabilities Development Command Weapons and Materials Research Directorate, Aberdeen Proving Ground, Aberdeen, MD 21005, USA

**Keywords:** additive manufacturing, rough surfaces, partial machining, WAAM 18Ni 250 Maraging steel, durability, crack growth

## Abstract

It is now well-known that the interaction between surface roughness and surface-breaking defects can significantly degrade the fatigue life of additively manufactured (AM) parts. This is also aptly illustrated in the author’s recent study on the durability of wire and arc additively manufactured (WAAM) 18Ni 250 Maraging steel specimens, where it was reported that failure occurred due to fatigue crack growth that arose due to the interaction between the surface roughness and surface-breaking material defects. To improve the durability of an AM part, several papers have suggested the machining of rough surfaces. However, for complex geometries the fully machining of the entire rough surface is not always possible and the effect of the partial machining on durability is unknown. Therefore, this paper investigates if partial machining of WAAM 18Ni 250 Maraging steel surfaces will help to improve the durability of these specimens. Unfortunately, the result of this investigation has shown that partial machining may not significantly improve durability of WAAM 18Ni 250 Maraging steel specimens. Due to the order of surface roughness seen in WAAM 250 Maraging steel, the improvement to durability is only realized by full machining to completely remove the remnants of any print artefacts.

## 1. Introduction

**Figure 1:**
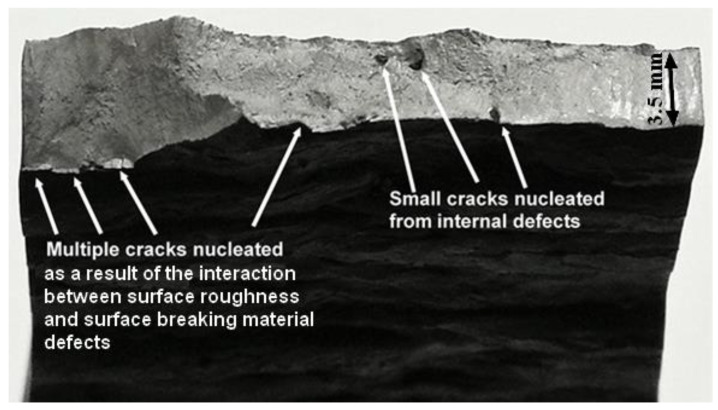
Picture of the failure surface of a WAAM 18Ni 250 Maraging steel test showing multiple cracks nucleating due to the interaction between surface roughness and surface-breaking material discontinuities, but little cracking associated with internal pores/voids.

**Figure 2:**
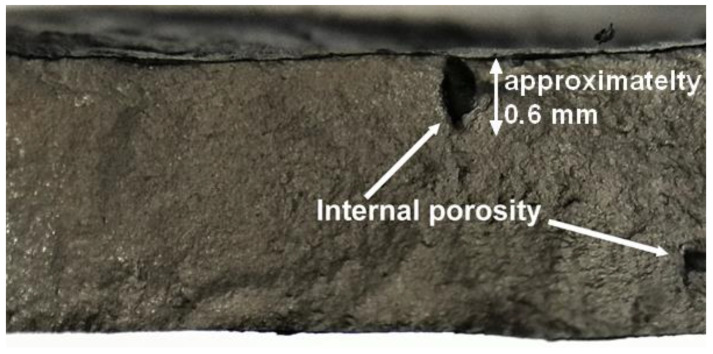
A close-up view of the failure surface shown in Figure 2 showing minimal crack growth associated with relatively large internal porosity/voids.

Practitioners in the field of additively manufactured structures have long been aware [1,2,3,4,5,6,7,8,9,10,11,12,13,14,15,16,17,18,19] that the interaction of surface-roughness with surface-breaking material discontinuities and surface-breaking porosity/lack of fusion can significantly degrade the durability and damage tolerance (DADT) of an additively manufactured part. Indeed, ref. [7] concluded that the fatigue life of AM Ti-6Al-4V parts built using either electron beam melt or direct metal laser sintering was determined by surface roughness effects (a picture illustrating how surface-breaking cracks can develop as a result of surface-breaking material discontinuities/defects is shown in Figure 1). The author’s recent paper [1] on the durability of WAAM 18NI 250 Maraging steel specimens tested with their surfaces left in the “as built” condition aptly illustrated this phenomenon in that the specimens failed due to the interaction between the rough-surface and surface-breaking material discontinuities. Although not reported in [1], this test program also found that the presence of large, wholly contained, internal voids/porosity and large near-surface voids did not result in failure. Examples of this are shown in Figure 1 and Figure 2. In each case, it was found that there was little crack growth from these internal and near-surface internal voids/porosity. Indeed, this observation is consistent with the seminal finding reported by Schijve [20], and subsequently confirmed in [21,22,23,24], that for conventionally built parts internal cracks grow much slower than the surface-breaking cracks.

Whereas [17,18,19] suggested that machining the rough surfaces may improve the DADT of an AM part, the studies presented in [2,15] suggested that partial machining may be of little benefit. However, the analysis presented in [2] examined an idealized (rough) surface where the surface roughness had a wave-like (sinusoidal) pattern. Consequently, noting that:

- The United States Air Force (USAF) MIL-STD-1530D [25] states that the certification of a load-bearing part must be based on analysis, and that the role of testing is to validate the analysis.
- Ref. [1] revealed that the durability and the associated crack growth histories of WAAM 18Ni 250 Maraging steel specimens with rough surfaces could be predicted in a fashion that was consistent with the linear elastic fracture mechanics approach mandated in the United States Air Force (USAF) MIL-STD-1530D.

The present paper addresses the question: Since failure of the WAAM 18Ni 250 Maraging steel specimens, which were built by Solvus Global in Worcester, in the United States of America (USA), in [1] was due to the interaction between surface-roughness and surface-breaking materials discontinuities, would partial machining of the surface help to improve the durability of these WAAM 18Ni 250 Maraging steel specimens?

Here it should be stressed that in contrast to the author’s prior study [2], the analysis reported in the present paper is based on actual (measured) surface roughness and uses the same crack growth equation that has been shown [1] to predict crack growth in specimens with as-built surface roughness.

## 2. Materials and Methods

As stated in the introduction, this paper is motivated by the research gap that exists when attempting to certify WAAM parts in accordance with the requirements inherent with USAF MIL-STD-1530D and USAF Structures Bulletin EZ-19-01 and the linear elastic fracture mechanics-based building block approach mandated in USAF MIL-STD-1530D. Furthermore, as mandated in MIL-STD-1530D certification requires the use of linear elastic fracture mechanics to predict durability. This approach is mandated both for WAAM parts in the as-built state, and for WAAM built parts that have had their rough surfaces partially machined. It should also be noted that Section 5.3 of MIL-STD-1530D states that analysis is the key to certification, and that the role of testing is to validate/correct the analysis. However, to the best of the author’s knowledge, other than the authors previous paper [1], there are no publicly available papers that have shown an ability to predict the durability, and the associated crack growth history, of WAAM steel specimens. It should be stressed that this is an essential step in the building block approach to certification, and is required before the analysis can assess the effect of partial machining. Consequently, this paper builds on the analysis methodology validated in [1] for predicting the durability of WAAM 18Ni 250 Maraging steel specimens to assess the effect of partial machining on WAAM 18Ni 250 Maraging steel specimen that was analyzed, in its “as-built” state, in [1].

For the sake of completion, it should also be noted that for the specimen with a rough surface analyzed in [1], the surface topography was first measured using an Artec3D Leo laser scanner (Santa Clara, CA, USA) that has a 3D point accuracy of approximately 0.1 mm. Consequently, as in [1], for the various durability analyses presented in this paper, the surface topography measurements obtained in [1] were first used to create a three-dimensional solid model. This CAD model was then auto-meshed to produce a three-dimensional finite element model of the particular specimen under consideration.

In the various crack growth analyses presented in this paper, the analyses began by assuming an initial crack size that was taken from the experimental measurements given in [1]. The stress intensity factor (*K*) solutions around this initial crack were determined, as per [1], using the multi-crack finite element analysis program developed as part of the US Federal Aviation Aging (FAA) Aircraft Program [26,27,28] and the stress field associated with the corresponding uncracked finite element model. The increment in the crack size (*da/dN*) around a given crack was then computed using the small crack growth equation for this material given in [1], viz:

*da/dN* = 2 × 10^−10^ ((Δ*K* − 0.1)/√(1 − *K*_max_/150))^2.0^ (1)

here Δ*K* = *K*_max_ − *K*_min_, where *K*_max_ and *K*_min_ are the maximum and minimum values of *K*, in a given cycle (*N*), and “*a*” is the crack length. Armed with this knowledge, a new crack size and shape is then determined. This process was repeated until fatigue failure, i.e., until at some point around the crack front the value of *K*_max_ exceeded the cyclic fracture toughness value of the material, which has a value of 150 MPa √m. An alternative implementation of this approach, which uses standard finite element analysis rather than the alternating finite element approach to compute the stress intensity factors around the crack, is now available in the commercial finite element programs ABAQUS^®^, NASTRAN^®^, and ANSYS^®^ via version 9 of the Zencrack^®^ software module [29].

The twenty journal papers referenced in this paper are listed in either the World of Science and/or SCOPUS. The book chapter referenced is available on the Elsevier website. The three United States (US) Government references related to the Department of Defence certification requirement are publicly available, and their web addresses are given. Keywords used in the search for these references were: Additive manufacturing, surface roughness, crack growth, and DADT.

## 3. On the Effect of Rough Surfaces on Durability

**Figure 3:**
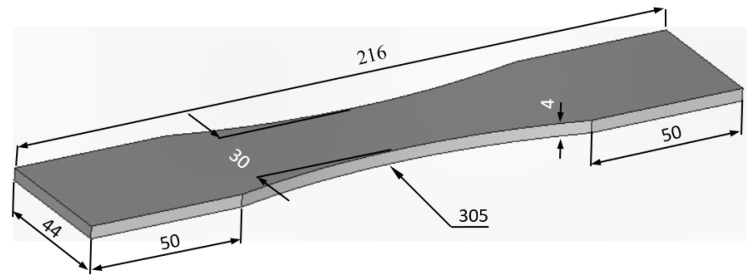
The geometry of the smooth surface dogbone specimen.

**Figure 4:**
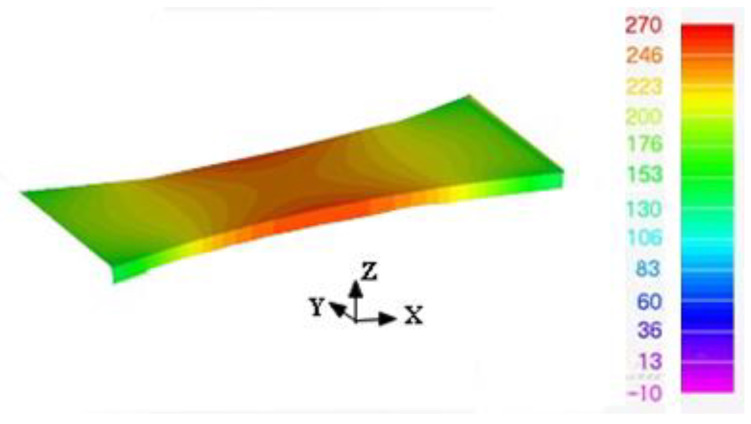
The computed stress field in the fine mesh finite element model at a remote load of 29 kN. The stress units in the picture are in MPa.

Recently, a paper [1] focused on the ability to compute the effect of the rough surfaces on the durability of as-built WAAM 18Ni 250 Maraging steel. However, it did not address the reduction in performance (durability) due to the rough surfaces. To illustrate, this let us first consider a 4 mm thick dogbone specimen with the same plan view as the specimen analyzed in [1], but with a uniform thickness of 4 mm, see Figure 3. In other words, the specimen has a smooth surface. As in [1], the specimen geometry was auto-meshed to produce both a fine and a coarse (finite element) mesh. The fine mesh consisted of approximately 45,936 21-noded iso-parametric elements and 211,085 nodes. The coarse mesh consisted of approximately 29,568 21-noded iso-parametric elements and 136,189 nodes. The difference in stress field between these two finite element models, at an applied load of 29 kN, was less than 0.5%. The stress field associated with the fine mesh is shown in Figure 4.

**Figure 5:**
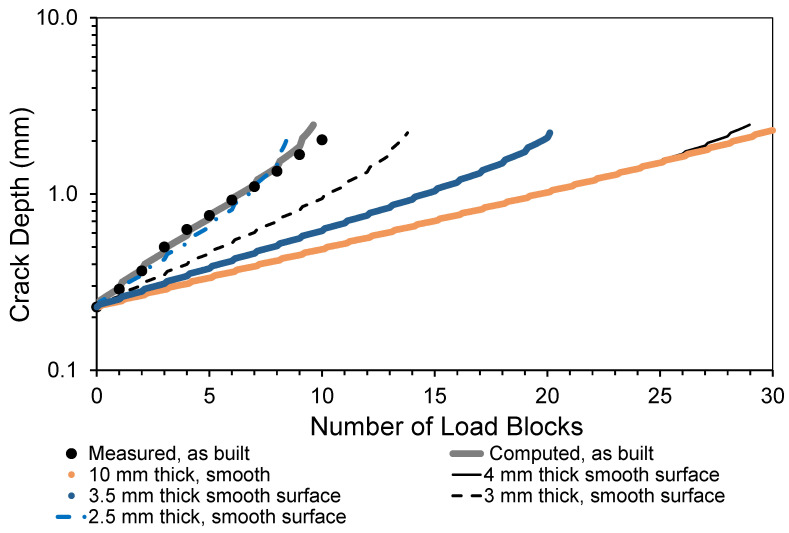
The measured and computed crack depth curves for a surface-breaking crack that is 0.228 mm deep and has a tip-to-tip length of 0.68 mm.

**Figure 6:**
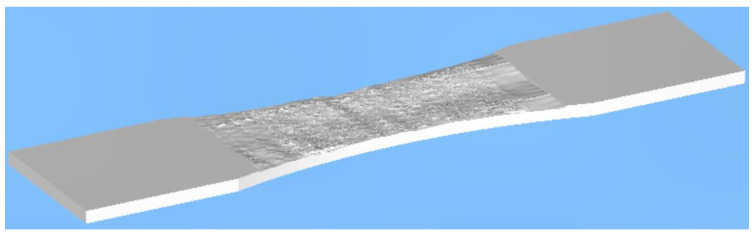
A typical CAD model with the surface roughness as measured in [1]. The in-plane dimensions are as shown in Figure 3.

As in [1], the analysis assumed an initial surface-breaking semi-elliptical crack that was 0.228 mm deep and had a tip-to-tip surface length of 0.680 mm and was located in the same position reported in [1], i.e., it was near the center of the specimen. This crack size was taken from fractography measurements associated with the failed specimen analyzed in [1]. Noting that, as can also be seen in Figure 5, the measured and predicted crack growth histories reported in [1], for the specimen with a rough “as-built” surface, were in excellent agreement, we used the same analysis approach to examine the durability of this 4 mm thick dogbone specimen. Furthermore, this “smooth surface” specimen was assumed to be subjected to the same repeated load block as in [1], with each load block consisting of 1200 cycles at *R* = 0.1, and 8000 cycles at *R* = 0.5. As in [1], the maximum load in the load block was held constant at *P_max_* = 29 kN (here the term *R* is the ratio of the minimum applied load divided by the maximum applied load). As previously mentioned, the durability analysis was performed using the small crack growth equation for this material given in [1], namely Equation (1). The resultant computed crack growth history is shown in Figure 5, together with that associated with the measured and computed histories given in [1] for the specimen with a rough surface (for the sake of, completion it should be noted that, for the specimen with a rough surface analyzed in [1], the surface topography was first measured using an Artec3D Leo laser scanner (Santa Clara, CA, USA) that has a 3D point accuracy of approximately 0.1 mm. The surface topography measurements were then used to create a three-dimensional solid model, which was then auto-meshed to produce a three-dimensional finite element model of the specimen, see Figure 6).

To study the effect of specimen thickness, the analysis was repeated for specimens with the same plan view and remote stress, but were either 2.5, 3, 3.5 or 10 mm thick, see Figure 5. Here we see that the effect of the surface roughness is to significantly increase the rate of crack growth in the specimen analyzed in [1], in comparison to the 4 mm thick specimen with a smooth surface. It is also seen that the crack growth rate associated with the specimen configuration tested in [1] is similar to that of a specimen that had a uniform thickness of 2.5 mm thick and had an initial crack that was 0.228 mm deep and had a tip-to-tip surface length of 0.680 mm.

**Figure 7:**
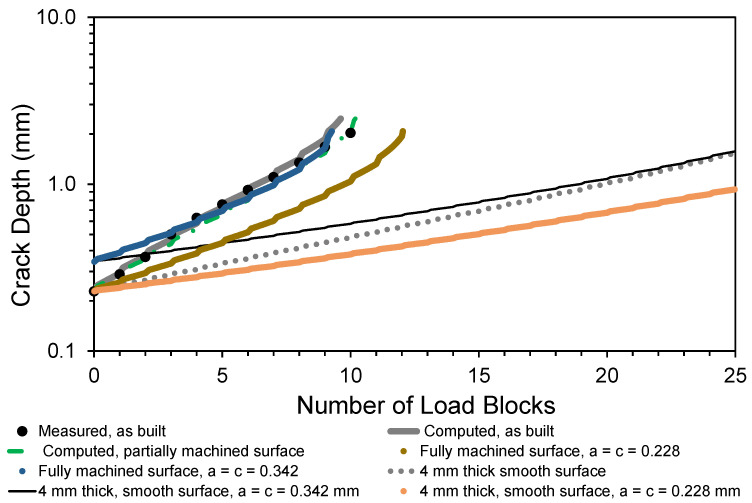
The measured and computed crack depth curves shown in Figure 5, together with the corresponding curve obtained for the partially machined surface and a specimen with a fully machined surface with a semi-circular surface-breaking crack.

To further highlight the effect of the rough surface on the life of the test specimen, Figure 7 presents a comparison of measured and computed crack growth histories given in [1] with the computed crack growth histories for a 4 mm specimen with a smooth surface and either a 0.228 mm radius or a 0.342 mm radius semi-circular surface-breaking crack.

## 4. Analysis of the Effect of Partial Machining on Durability

Having seen that the surface roughness would appear to significantly reduce the durability of the specimen, let us next address the question: Will partial machining of the surface help to improve the durability of WAAM built 18Ni 250 Maraging steel specimens?

To this end, the finite element model of the specimens developed [1] for the as-built WAAM 18Ni 250 Maraging steel specimens was modified such that 0.72 mm of the (upper) as-built surface was removed (this equates to roughly one-half of the maximum height of the as-built surface).

**Figure 8:**
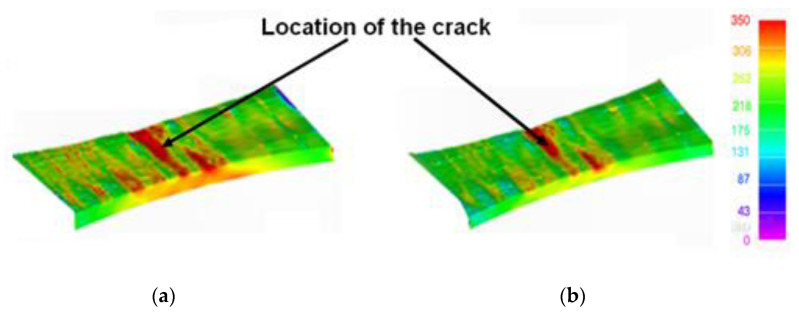
The stress fields in the (**a**) as-built, and (**b**) partially machined specimens.

This “partially machined” model was assumed to be subjected to the same repeated load block spectrum, and a comparison between the stress states in the “as-built” and the “partially machined” states is given in Figure 8a. As previously, the initial crack size assumed in the analysis was 0.228 mm deep and had a tip-to-tip surface length of 0.680 mm. Since the modification of the CAD model to reflect partial machining of the surface did not result in the region where the surface-breaking crack was located being removed, the location of this surface-breaking crack remained as in [1], i.e., on the rough surface near the center of the specimen. Furthermore, as in [1], the crack growth analysis used Equation (1).

This analysis gave a durability of approximately 10.0 load blocks. A comparison between the computed crack growth history for specimens in both the as-built and the partially machined state is also given in Figure 7. Here we see that for the measured initial crack size, there is little difference between the crack growth histories associated with the as-built and the partially machined specimens. The reason for this becomes clear upon inspecting Figure 8, where we see that partial machining did not significantly improve the stress field at the critical location.

### 4.1. Thickness Effects

**Figure 9:**
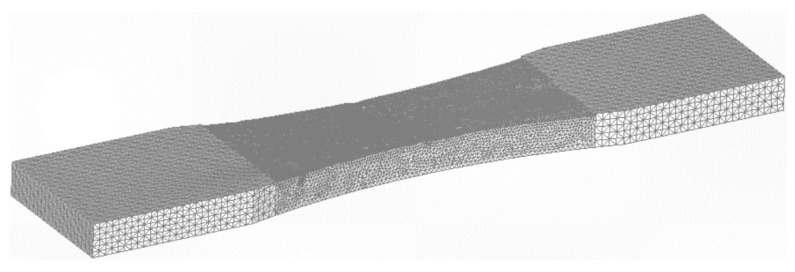
The coarse mesh, 300,233 ten nodded iso-parametric tetrahedral elements and 437,380 nodes.

**Figure 10:**
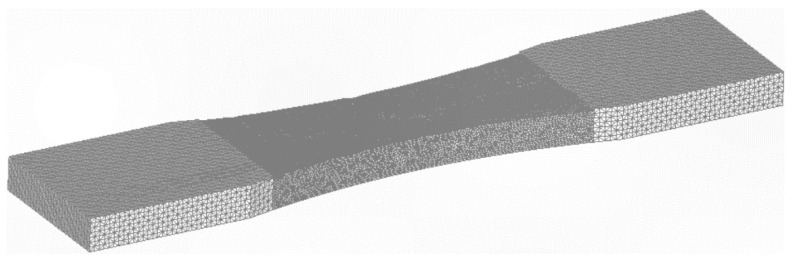
The fine mesh, 609,768 ten nodded iso-parametric tetrahedral elements and 876,866 nodes.

The question now arises as to what would happen if the specimen was thicker? To investigate this question, the analysis was repeated with the thickness increased to 10 mm. To ensure that the remote stress was the same as for the 4 mm thick specimen, the load was increased to 72.5 kN. Analyses were again performed for 10 mm thick specimens with the same as-built and partially machined surface profiles, and for a 10 mm thick specimen with a smooth surface. In each case, to establish convergence, the specimen geometry was auto-meshed to produce both a fine and a coarse (finite element) mesh. For example, in the case of the partially machined specimen, the coarse mesh consisted of approximately 300,233 ten nodded iso-parametric tetrahedral elements and 437,380 nodes, see Figure 9. The fine mesh consisted of approximately 609,768 ten nodded iso-parametric tetrahedral elements and 876,866 nodes, see Figure 10. The difference in stress field between these two finite element models was less than 1.6%. In all cases, the durability analyses used the finer of the two meshes.

**Figure 11:**
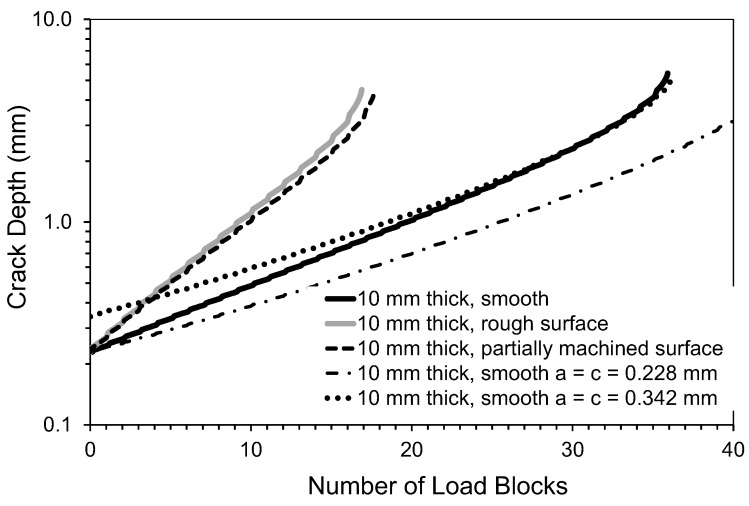
The computed crack depth curves for a 10 mm thick specimen.

Figure 11 presents a comparison between the computed crack growth histories for 10 mm thick specimens in both the as-built and the partially machined state and for a 10 mm thick specimen with a smooth surface. Since MIL-STD-1530D [25] and the United States Joint Services Structural Guideline JSSG2006 [30] suggest that, for a conventionally manufactured part, the aspect ratio of the initial crack should be one, i.e., *c*/*a* = 1, analyses were performed with the initial crack size assumed being either:

(i) A 0.228 mm deep semi-elliptical surface-breaking crack with a tip-to-tip length surface length of 0.680 mm.
(ii) A 0.228 mm radius semi-circular surface-breaking crack.
(iii) A 0.342 mm radius semi-circular surface-breaking crack.

Here we see that, despite the increased thickness, partial machining only resulted in a relatively small increase in durability. Furthermore, regardless of the assumptions used in the analysis of the durability of the smooth specimen, the durability of the partially machined specimen was significantly less than that of a specimen with a smooth surface. This finding, when taken in conjunction with the results presented in [2,15] and the above sections of this paper, adds support to the conclusion that partial machining may not be particularly effective in improving the durability of these WAAM-built steel specimens.

### 4.2. A Fully Machined Surface

The question now arises: What would happen if the rough surface was machined entirely flat? For the rough surface specimen discussed in [1] and Section 3, this would result in an approximately 2.6 mm thick specimen. The resultant crack growth history, for this “fully machined” specimen with the same size initial crack, is also shown in Figure 7. Unfortunately, machining the specimen flat increases the stress in the specimen. Consequently, as a result of the subsequent increase in the stress in the section, the resultant crack growth history is not significantly improved, see Figure 7.

The analysis of the fully machined specimen was repeated assuming an initial 0.228 mm radius and a 0.342 mm radius semi-circular surface-breaking crack. The results of this analysis are also shown in Figure 7. Here, we see that for a 0.228 mm radius semi-elliptical crack the life of the fully machined part, as computed using the aspect ratio *c*/*a* = 1 recommended in [25,30] for a conventionally manufactured specimen, is approximately 20% greater than that computed for the as-built part with the measured 0.228 mm deep and 0.680 mm tip-to-tip length crack. However, if the radius of the semi-elliptical crack is 0.342 mm then the life of the fully machined part is essentially the same as that computed for the as-built part.

Here it should be remembered that this specimen was only (nominally) four mm thick. Consequently, removing approximately 1.4 mm to ensure a smooth surface has a significant effect on the stress in the specimen. The increase in stress would be less for thicker specimens. As such, the benefit of completely machining away the surface roughness may need to be studied on a case-by-case basis.

## 5. Conclusions

The author’s prior study into the durability of wire and arc additively manufactured (WAAM) 18Ni 250 Maraging steel specimens revealed that failure occurred due to the interaction between the surface roughness and surface-breaking material discontinuities. The present paper has revealed that in these tests, there was little crack growth from internal and near-surface, internal voids/porosity. This observation is consistent with that reported in tests with internal voids/porosity in conventionally manufactured parts. As a result, the present paper has addressed the questions:

(i) How severely does the rough “as-built” surface degrade the durability of the specimen?
(ii) Will partial machining of the surface help to improve the durability of WAAM built steel specimens?

Unfortunately, the results of this investigation suggest that:

(a) Surface roughness of the order of that seen in the WAAM 18Ni 250 Maraging steel specimens significantly degrades its durability.
(b) Partial machining of the rough surface may not significantly improve durability.
(c) The benefit of fully machining a rough surface is best suited to relatively thick specimens where the loss of material due to machining does not significantly increase the stress in the remaining material.

## Data Availability

The data are not yet publicly available due to the ongoing nature of this project. The data will be available on completion of the study.
